# Sensory Processing Profiles and Learning Potential in Autism: A Longitudinal Study of Cognitive Development in Preschoolers

**DOI:** 10.3390/jintelligence14070132

**Published:** 2026-07-01

**Authors:** María Auxiliadora Robles-Bello, Francisca Barba-Colmenero, Jonathan Vinicio Camino-Alarcón, Nieves Valencia-Naranjo

**Affiliations:** Department of Psychology, University of Jaén, 23071 Jaén, Spain; fbarba@ujaen.es (F.B.-C.); nnaranjo@ujaen.es (N.V.-N.)

**Keywords:** sensory processing, dynamic assessment, preschoolers, cognitive skills, autism spectrum disorder

## Abstract

Background: Sensory processing differences are highly prevalent in children with autism spectrum disorder (ASD) and may shape how they interact with their environment and acquire new knowledge. The present study investigated developmental changes in learning potential and cognitive functioning in preschool children with ASD presenting a sensory seeking profile with typical sensitivity. In addition, the study examined which components of early learning potential predicted intellectual functioning two years later. Methods: A longitudinal design was employed with 44 preschool boys with ASD assessed at ages four and six. The potential for learning through dynamic assessment, intelligence and sensory processing patterns was assessed. Paired sample *t*-tests were conducted to examine developmental changes, and hierarchical regression analyses were used to identify predictors of intellectual functioning at age six. Results: Significant improvements were observed in general cognitive ability, classification ability, and perspective-taking skills across the two-year period. Regression analyses revealed that visual memory, sequential pattern completion, classification ability, and perspective taking significantly predicted intellectual functioning at age six, jointly explaining 51% of the variance in IQ scores. Conclusions: The findings suggest that visuospatial processing and pattern-based reasoning may play a central role in cognitive development among preschool children with ASD who exhibit active sensory engagement.

## 1. Introduction

Autism spectrum disorder (ASD) is a neurodevelopmental condition characterized by persistent differences in social communication and interaction, together with restricted or repetitive patterns of behavior and interests (American Psychiatric Association, 2014). One of the most widely recognized features of autism is the remarkable heterogeneity observed in cognitive functioning, behavioral patterns, and developmental trajectories. (Lord et al., 2022). Rather than representing a uniform profile of strengths and difficulties, individuals with ASD often present highly specific cognitive and perceptual patterns that may shape the way they interact with and learn from their environment (Valeri et al., 2020).

Understanding how children with ASD learn is therefore a central challenge for both developmental research and early educational intervention (Zuckerman et al., 2021). Traditional psychometric assessments primarily measure current performance and may not fully capture the child’s capacity to benefit from instruction or mediated support. For this reason, increasing attention has been directed toward dynamic assessment approaches, which aim to evaluate the learner’s potential for cognitive change rather than static levels of performance (Haywood & Lidz, 2007; Musci & Brenlla, 2017).

The application of dynamic assessment in the field of autism has emerged as a response to the limitations of static IQ tests, which often underestimate the cognitive potential of these children due to social and communicative barriers. However, research in this area has shown inconsistencies; while some studies suggest that the social nature of the mediation phase can facilitate better performance, others warn that for many children with ASD, the increased social interaction required during the test can also be a source of anxiety or sensory overload. Furthermore, the lack of control over the child’s sensory profile in prior research may explain why some children benefit from mediation while others do not. Our study seeks to address this gap by exploring whether a specific sensory phenotype—the Sensory Seeking profile—is more receptive to clinical interaction and mediation.

Dynamic assessment is conceptually rooted in Vygotsky’s sociocultural theory, particularly in the concept of the Zone of Proximal Development (ZPD), which refers to the difference between what a learner can achieve independently and what can be achieved with guidance from a more knowledgeable partner (Vygotsky, 1978). Within this framework, learning is understood as a socially mediated process in which cognitive development can be fostered through structured interaction and support (Daneshfar & Moharami, 2018). Feuerstein later expanded this perspective through the concept of Mediated Learning Experience (MLE), emphasizing that cognitive functioning is modifiable when individuals are exposed to intentional mediation during learning situations (Feuerstein et al., 1986).

Dynamic assessment procedures are therefore designed to examine cognitive modifiability, that is, the degree to which a learner can improve performance when provided with guided instruction (Tzuriel & Caspi, 2017). This approach is particularly relevant for populations with atypical developmental trajectories, such as children with autism, where conventional assessments may underestimate learning potential (Gómez-Pérez et al., 2020; Valencia-Naranjo & Robles-Bello, 2017).

At the same time, another domain that has received increasing attention in autism research is sensory processing. Sensory processing refers to the way in which the nervous system detects, organizes, and interprets sensory information from the environment in order to generate adaptive behavioral responses (Miller et al., 2007). Atypical sensory processing is extremely common in ASD, with studies suggesting that up to 90% of autistic individuals show differences in sensory reactivity across one or more modalities (Crasta et al., 2020; Gonçalves & Monteiro, 2023).

Importantly, sensory processing patterns may not only influence behavior and emotional regulation but also shape the ways in which children explore and learn from their environment (Stevenson et al., 2021). According to Dunn’s sensory processing framework, individuals differ in their neurological thresholds for sensory input and in the strategies they use to regulate sensory experiences (Dunn, 2016). These differences lead to distinct sensory response patterns, including sensory seeking, sensory sensitivity, sensory avoidance, and low registration (Fernández-Pires et al., 2020).

Among these patterns, sensory seeking behaviors are characterized by active exploration of sensory stimuli and a tendency to seek increased sensory input. In early childhood, such behaviors may be associated with heightened engagement with visual, proprioceptive, or movement-based experiences. From a developmental perspective, this exploratory orientation could potentially support certain forms of learning, particularly those involving pattern detection, visuospatial processing, and interaction with structured sensory environments (Stevenson et al., 2021; Zippert et al., 2021).

Despite growing interest in sensory processing in autism, relatively little research has examined how specific sensory profiles may be associated with different cognitive learning pathways (Camino-Alarcón et al., 2024).

Investigating this relationship may provide valuable insights into the mechanisms through which sensory experiences contribute to cognitive development in autism (Bazelmans et al., 2021; Dellapiazza et al., 2020). Moreover, examining learning potential within relatively homogeneous sensory profiles may allow researchers to identify more specific developmental patterns that might otherwise remain hidden in highly heterogeneous samples (Bazelmans et al., 2021).

The present study adopts this perspective by focusing specifically on preschool children with ASD presenting a sensory seeking profile combined with moderate to high sensory sensitivity. Rather than representing a methodological limitation, this selection was intended to increase sample homogeneity and facilitate the identification of potential learning pathways associated with this sensory profile (Dunn, 2016; Fernández-Pires et al., 2020).

Most studies addressing sensory processing in ASD have focused on behavioral outcomes, adaptive functioning, or sensory-related difficulties (Dellapiazza et al., 2020; Robain et al., 2020), while the potential relationship between sensory experiences and cognitive learning pathways remains underexplored (Stevenson et al., 2021).

In particular, few studies have adopted a longitudinal perspective to investigate how early learning potential interacts with sensory processing characteristics to predict later intellectual outcomes (Luković et al., 2022). Understanding these relationships is particularly important during the preschool years, a developmental period marked by high neural plasticity and rapid cognitive change (Lord et al., 2022).

Dynamic assessment approaches provide a promising framework for addressing this gap. Unlike traditional psychometric assessments that capture only static levels of performance, dynamic assessment evaluates the child’s capacity to benefit from mediated learning experiences (Haywood & Lidz, 2007; Kee, 2016). This perspective allows researchers to identify modifiable cognitive processes that may contribute to later development (Tzuriel & Caspi, 2017; Valencia-Naranjo & Robles-Bello, 2020).

The present study addresses this gap by integrating sensory processing theory with dynamic assessment of learning potential in a longitudinal design. Specifically, the research focuses on preschool children with ASD presenting a sensory seeking profile with typical sensitivity, a pattern characterized by active sensory exploration combined with relatively preserved self-regulation. Examining a relatively homogeneous sensory subgroup may help reveal more specific cognitive learning pathways that are often obscured in heterogeneous ASD samples.

The synthesis of these frameworks provides a coherent explanatory model for this study: Dunn’s sensory model explains how children with ASD perceive and interact with environmental stimuli, which, according to Feuerstein (1980), is the prerequisite for a ‘Mediated Learning Experience.’ Specifically, we argue that a sensory seeking profile acts as an intrinsic motivational driver that pushes the child toward the ‘Zone of Proximal Development’ (Vygotsky, 1978). By actively seeking sensory input, these children create more frequent opportunities for cognitive mediation, potentially leading to the structural cognitive modifiability that our dynamic assessment (EHPAP) aims to quantify. Thus, sensory processing is not merely a co-occurring symptom, but a foundational mechanism that may facilitate or hinder the expression of learning potential.

In this study, learning potential is operationally defined as the child’s capacity to benefit from adult mediation to solve problems that they could not solve independently. This concept, rooted in the ‘propensity for change,’ goes beyond a static score; it represents the dynamic interplay between the child’s baseline cognitive resources and their responsiveness to structured pedagogical cues (Feuerstein et al., 2002). By quantifying this modifiability, we aim to capture the latent cognitive abilities that remain ‘hidden’ in traditional IQ tests due to the specific social and sensory challenges of ASD.

The choice to focus on the sensory seeking profile is based on the theoretical assumption that a high drive for environmental interaction may serve as a precursor to more effective mediated learning experiences, providing a clearer window into how sensory-cognitive pathways develop in ASD. This choice is supported by evidence suggesting that ‘Seekers’ often demonstrate higher levels of social engagement and environmental mastery compared to ‘Avoiders’ or ‘Low Registration’ profiles (Dunn, 1997). Recent studies have also indicated that children who actively seek sensory input may possess a more proactive learning style, which is a critical prerequisite for cognitive modifiability (Dellapiazza et al., 2020). Therefore, focusing on this subgroup allows for a clearer window into how specific sensory-cognitive pathways develop in ASD, reducing the noise typically associated with more avoidant sensory phenotypes.

It is important to clarify that we do not propose a unidirectional causal pathway where sensory processing dictates cognitive outcomes. Rather, we suggest that sensory profiles and learning potential engage in a reciprocal interaction. A Sensory Seeking profile may act as a facilitating mechanism by increasing the child’s active exposure to learning opportunities, providing the necessary ‘raw material’ for cognitive modifiability to operate. Therefore, our study explores these sensory-cognitive associations as integrated components of a developmental system.

In distinguishing between the impacts of sensory processing, it is crucial to separate sensory-driven behaviors from their cognitive implications. While sensory seeking is often documented as a behavioral manifestation (e.g., motor agitation or tactile exploration), in this study, we focus on its role as a cognitive mediator. We argue that beyond the observable behavioral ‘noise,’ the sensory seeking profile reflects a specific mode of environmental engagement that determines how a child selects, filters, and prioritizes information during learning tasks. By isolating the sensory seeking profile, we aim to observe how this behavioral drive translates into a cognitive advantage—or barrier—within the structured learning environment of dynamic assessment.

Therefore, the objectives of the study were: (1) to examine developmental changes in learning potential and cognitive functioning between ages four and six; (2) to identify which components of early learning potential predict later intellectual functioning; and (3) to characterize the specific cognitive learning patterns associated with this high sensory engagement profile.

Regarding the third objective, we hypothesized that the sensory seeking profile would be positively associated with higher cognitive modifiability. Specifically, we expected these children to demonstrate greater responsiveness to mediation and a more efficient transition from assisted to independent problem-solving due to their proactive environmental engagement style.

## 2. Materials and Methods

### 2.1. Participants

The participants were 44 preschool boys with ASD aged 4–6 years. The recruitment was primarily conducted at a single Early Childhood Intervention (ECI) center where the lead researcher was based, ensuring high consistency in the administration of the assessments. To reach the final sample size, a small subgroup of participants (*n* < 10) was recruited from two other nearby centers within the same city. This approach ensured that all children shared a similar geographical and socio-educational context while maintaining the reliability of the data, as all longitudinal evaluations (at ages 4 and 6) were performed by the same trained examiner.

All participants were male, meaning the findings are limited to this gender. At the first timepoint, the children were 4 years old and in the second year of kindergarten (M = 4.2, SD = 2.3). At the follow-up assessment (2 years later), the children were 6 years old (M = 6.7, SD = 5.1). This is a significant limitation, as female phenotypes in ASD often involve different sensory reactivity and social camouflage strategies (Hull et al., 2017; Lai et al., 2015).

Participants were selected based on a predominant sensory seeking pattern. The primary inclusion criterion for the ASD group was the presence of a sensory seeking profile with Typical Sensitivity, defined by obtaining scores in the sensory seeking quadrant (Percentile > 60) and scores within the typical range in the Sensory Sensitivity and Low Registration quadrants (Percentiles near 45 and 35, respectively) of the Sensory Profile–2 (Dunn, 2014). This pattern was specifically accompanied by high scores in the Visual and Movement sensory sections (Percentile > 60), as detailed in Table 1. These children are characterized by actively seeking sensory stimuli, especially visual or proprioceptive, while maintaining typical self-regulation and functionality in the classroom. They may also exhibit some hyperactivity in noisy contexts or when overloaded with unwanted stimuli, which can be modulated with minimal support.

All participants received intervention within the same early intervention program, ensuring a consistent therapeutic context. This consistency minimized the potential confounding effect of different intervention methodologies on the observed longitudinal outcomes.

Regarding the concern about the ‘average profile’ in Table 1, we agree that individual variability is a hallmark of ASD. To address this, we have ensured that the Standard Deviations (SDs) are clearly reported for all measures to reflect this dispersion. Furthermore, the inclusion criteria were strictly applied at the individual level before aggregate scores were calculated, ensuring that each child in the study independently met the criteria for the ‘Sensory Seeking’ profile. This approach balances the need for group-level statistical analysis with the recognition of individual differences.

### 2.2. Instruments

The Escala de Habilidades y Potencial de Aprendizaje en Preescolares (EHPAP) (Calero et al., 2009) is for use with Spanish preschoolers aged between three and six years and is known in Spain. This assessment tool is designed to examine several cognitive processes that are closely related to early learning and problem-solving. The scale includes multiple tasks aimed at evaluating specific abilities such as categorization, auditory recall, visual memory, pattern sequencing, verbal planning, and the capacity to adopt another person’s perspective.

The EHPAP is a dynamic assessment tool grounded in Feuerstein’s theory of Mediated Learning Experience (MLE), which has extensive cross-cultural validation. Regarding its psychometric properties, the instrument has shown high internal consistency (Cronbach’s alpha > 0.85) and strong convergent validity with other measures of cognitive modifiability and intelligence in clinical populations with ASD within the Mediterranean context (Calero et al., 2009, 2017). While primarily utilized in Spanish-speaking cohorts, its focus on non-verbal problem-solving and standardized mediation levels minimizes linguistic bias, making it a reliable indicator of learning potential across different educational settings.

In the categorization task, children are asked to sort geometric figures according to different attributes, including color, shape, and size, allowing evaluators to observe how they organize information based on shared characteristics. The auditory memory task involves listening to a short story and later recounting it; performance is judged by considering how many details the child remembers and whether the events are reproduced in the correct order. The visual memory activity requires children to recall common objects previously presented visually, while also taking into account the memory strategies they use to support recall.

Another component of the scale is sequential pattern completion, in which children must identify and continue visual patterns. Scoring reflects both the number of sequences correctly completed and the child’s ability to justify or explain their choices. The perspective-taking task focuses on communicative and cooperative abilities: the child provides instructions to an adult who is drawing, and the evaluation considers how effectively verbal and non-verbal cues are used to guide the activity. Lastly, the verbal planning task examines children’s ability to describe how they would carry out a routine activity. In this case, the assessment takes into account whether the child outlines the necessary steps, demonstrates a coherent overall plan, and uses language that reflects sequencing or planning processes.

To assess cognitive functioning more broadly, the study also employed the Woodcock–Muñoz Battery III, which corresponds to the Spanish parallel version of the Woodcock–Johnson III assessment system (Muñoz-Sandoval et al., 2005). This battery can be administered to individuals ranging in age from early childhood to adulthood. It comprises a set of tests designed to measure cognitive abilities as well as academic skills. The cognitive component is grounded in the Cattell–Horn–Carroll (Gf–Gc) theoretical model, which conceptualizes intelligence as a combination of several broad cognitive capacities. Among these abilities are long-term retrieval processes, short-term memory, processing speed, auditory and visual processing, comprehension and acquired knowledge, and fluid reasoning. Results from these measures can be combined to generate an overall general intellectual ability index (g), which was used in the present study as an indicator of participants’ global cognitive functioning. Specifically, the Brief Intellectual Ability (BIA) score was utilized, derived from three subtests: Verbal Ability (Test 1: Woodcock–Muñoz Vocabulary), Thinking Ability (Test 2: Spatial Relations), and Cognitive Efficiency (Test 3: Concept Formation). To ensure longitudinal consistency, the same version and normative scales were strictly maintained across both timepoints (age 4 and age 6).

In addition, sensory processing patterns were evaluated using the Sensory Profile–2 School Companion (Dunn, 2016), a standardized questionnaire widely used in educational contexts. This instrument is based on Dunn’s sensory processing framework, which explains individual differences in sensory behavior through the interaction between neurological sensitivity thresholds and the strategies individuals use to regulate their responses. Within this model, four characteristic patterns of sensory response are typically identified: low registration, sensory seeking, sensory sensitivity, and sensory avoidance.

The school version of the questionnaire is completed by teachers and gathers information about how students respond to stimuli across multiple sensory domains, including auditory, visual, tactile, vestibular, proprioceptive, and oral systems. In addition to sensory responses, the questionnaire addresses behavioral aspects linked to attention, emotional and behavioral self-regulation, and social participation in classroom activities. Each item is rated using a five-point Likert-type scale, reflecting the frequency with which a particular behavior is observed in the school setting. After scoring, results are converted into percentile values, allowing comparison with normative data and helping determine whether the child’s responses fall within expected developmental limits or deviate from typical patterns. The instrument has demonstrated strong psychometric properties, including high internal consistency (Cronbach’s alpha values between 0.80 and 0.95) and satisfactory construct validity. For this reason, it is commonly used in research and clinical practice, particularly in the evaluation of children with autism spectrum disorder (ASD), where it supports the development of tailored educational strategies and intervention plans. The data obtained from this instrument and used in the statistical analyses represent raw scores.

### 2.3. Data Design and Analysis

The study followed a longitudinal design with two measurement points (ages 4 and 6). Descriptive statistics were calculated for all study variables, including means and standard deviations. Prior to inferential analyses, the distributional properties of the data were examined to verify the assumptions required for parametric testing. To examine developmental changes in general cognitive ability and learning potential components (classification, auditory memory, visual memory, sequential pattern completion, perspective taking, and verbal planning), paired sample *t*-tests were conducted to compare scores obtained at age four and age six. Effect sizes were calculated using Cohen’s d to estimate the magnitude of developmental change.

To identify which components of learning potential predicted intellectual functioning at age six, a hierarchical multiple regression analysis was performed. Predictors were entered in theoretically meaningful blocks: (1) conceptual learning variables (classification and auditory memory), (2) visuospatial and pattern reasoning variables (visual memory and sequential pattern completion), and (3) sociocognitive and executive variables (perspective taking and verbal planning). This approach allowed evaluation of the incremental variance explained by each cognitive domain.

Prior to interpreting the regression models, the main statistical assumptions were examined. Multicollinearity was assessed using Variance Inflation Factor (VIF) and tolerance values. Normality of residuals was evaluated through the Shapiro–Wilk test and inspection of Q–Q plots. Homoscedasticity was examined by inspecting standardized residual plots and by conducting the Breusch–Pagan test. No substantial violations of these assumptions were observed.

All analyses were conducted using SPSS version 26 (Field, 2005) (IBM Corp., Armonk, NY, USA). To ensure the robustness of the longitudinal predictions, baseline IQ scores at age 4 were accounted for in the analysis. This allowed us to determine the unique contribution of the ‘Sensory Seeking’ profile to cognitive modifiability and later IQ at age 6, controlling for initial intellectual functioning. To address the potential risk of overfitting due to the sample size, predictors were selected based on strong theoretical relevance and observed effect sizes. Post hoc diagnostics, including the examination of residuals and collinearity, were conducted to confirm the stability and reliability of the regression model. Furthermore, the interpretation of results prioritized effect sizes to ensure practical significance, with post hoc power analyses confirming that the sample was sufficient to detect the medium-to-large effects observed in the sensory-cognitive relationship. Statistical **s**ignificance was set at *p* < .05.

Prior to the main analyses, data were screened for outliers and influential cases using Cook’s distance. No cases exceeded the threshold of 1.0, and both skewness and kurtosis were within acceptable ranges, ensuring that the regression results were not disproportionately influenced by individual cases.

### 2.4. Procedure

The sample consisted of children receiving services at a single Early Childhood Intervention (ECI) center, where they were enrolled in a developmental support program. Participation in the study required prior written consent from parents or legal guardians, obtained before the beginning of the evaluation process. After consent had been granted, the assessment protocol was implemented through individual sessions conducted with each child.

The research followed a longitudinal design with two measurement points. The baseline assessment was performed when the participants were four years old, and the same children were reassessed two years later, at six years of age, allowing the study to examine developmental changes across this period.

The study was formally approved by the Chair of the Ethics Committee of the Vice-Rectorate for Research at the University of Jaén (Spain) (Approval: MAR 18/12. TES) and Bioethics Committee for Research on Human Subjects of the Catholic University of Santiago de Guayaquil (Ecuador) (Approval: CEISH-UCSG.0153.01). Prior to the commencement of the study, written informed consent was obtained from the legal guardians of all participants. To ensure strict confidentiality and data protection, all individual datasets were fully anonymized by assigning a unique identification code (ID) to each participant; no personal identifiable information was retained during the analysis. Participants attended Early Childhood Intervention (ECI) centers for an average of 2 h per week, receiving multidisciplinary support that combined occupational therapy (sensory integration), speech therapy, and psycho-educational intervention.

## 3. Results

This section provides a concise and precise description of the experimental results, their interpretation, and the experimental conclusions that can be drawn.

To examine developmental changes between the two assessment points (ages 4 and 6), paired sample *t*-tests were conducted for general cognitive ability and for each component of learning potential assessed through the EHPAP. This approach was selected because the same participants were assessed at both timepoints.

The results indicated a significant increase in general cognitive ability across the two-year interval. Mean IQ scores increased from age four (M = 100.2, SD = 15.0) to age six (M = 106.7, SD = 20.6). The paired-sample *t*-test indicated that this difference was statistically significant (*t* (43) =2.28, *p* = .035), suggesting a moderate developmental improvement in intellectual functioning and learning potential during the preschool years (Table 2).

With respect to the EHPAP subscales, developmental patterns differed across cognitive domains: (1) Classification ability: Showed a statistically significant increase between the two assessment points, indicating progress in conceptual organization; (2) Perspective-taking ability: Increased significantly, suggesting improvements in sociocognitive coordination and communicative guidance; (3) Other processes: Sequential pattern completion and verbal planning showed small increases that did not reach statistical significance; (4) Memory: Auditory and visual memory remained relatively stable across the two-year period.

To examine which aspects of early learning potential were associated with later cognitive functioning, a hierarchical multiple regression analysis was conducted to predict IQ scores at age six. The six EHPAP subscales were entered as predictors in theoretically defined blocks: (1) Conceptual learning variables: Classification and auditory memory; (2) Visuospatial and pattern reasoning variables: Visual memory and sequential pattern completion; (3) Sociocognitive and executive variables: Perspective taking and verbal planning.

The distribution of standardized residuals did not significantly deviate from normality (Shapiro–Wilk W = 0.97, *p* = .41). Homoscedasticity was evaluated by examining scatterplots of standardized residuals against predicted values. The distribution of residuals appeared randomly dispersed around zero, indicating constant variance. In addition, the Breusch–Pagan test was not significant (χ^2^ = 5.12, *p* = .27), suggesting that the assumption of homoscedasticity was met. Diagnostics indicated that the predictors were not highly correlated. Tolerance values ranged between 0.59 and 0.71, while variance inflation factor (VIF) values ranged between 1.40 and 1.70, indicating that multicollinearity was not a concern in the regression model.

The regression was performed in three distinct steps (Table 3): (1) Conceptual learning variables (classification and auditory memory) were entered into the model. This model explained 18% of the variance in IQ, *R*^2^ = 0.18, F (2,41) = 4.47, *p* = .017. Within this model, classification emerged as a significant predictor, whereas auditory memory did not reach statistical significance. (2) Visuospatial and reasoning-related abilities (visual memory and sequential pattern completion) were introduced. This significantly improved the predictive power of the model, explaining an additional 24% of the variance in IQ (ΔR^2^ = 0.24, *p* < .01). In this model, visual memory emerged as the strongest predictor of cognitive ability, followed by sequential pattern completion. (3) Sociocognitive and executive processes (perspective taking and verbal planning) were included. This final model accounted for 51% of the variance in IQ, *R*^2^ = 0.51, Adjusted *R*^2^ = 0.44, F (6,37) = 6.42, *p* < .001.

In the final model, visual memory, sequential pattern completion, classification ability, and perspective taking were significant predictors of intellectual functioning at age six. In contrast, auditory memory and verbal planning did not contribute significantly to the prediction. These results suggest that visuospatial processing and pattern reasoning abilities play a central role in predicting cognitive outcomes in preschool children with high-functioning ASD.

## 4. Discussion

The present longitudinal study investigated developmental changes in learning potential and cognitive functioning in preschool children with autism spectrum disorder (ASD) characterized by a sensory seeking profile with typical scan-in-sensitivity (Dunn, 2016). Additionally, it examined which components of early learning potential predicted intellectual functioning two years later. Overall, the findings contribute to a growing body of research suggesting that cognitive development in autism is shaped not only by general developmental maturation but also by domain-specific cognitive strengths and by the interaction between sensory processing patterns and learning mechanisms (Lord et al., 2022; Miller et al., 2007).

First, the results revealed a significant increase in general cognitive ability between ages four and six. This pattern aligns with longitudinal studies indicating that early childhood represents a period of substantial neurocognitive plasticity in children with ASD, particularly when early intervention and structured educational environments are present (Robain et al., 2020; Zuckerman et al., 2021). This increase in IQ scores may reflect the rapid cognitive changes and biological maturation characteristic of this stage (Johnson, 2011), while also providing evidence of the cognitive modifiability of the sample. The observed effect size (*d* = 0.53) indicates a medium magnitude of change, suggesting that this improvement is clinically meaningful and represents a substantial gain in learning capacity. In this sense, the learning potential measured at age 4 serves as a foundation for later intellectual functioning, suggesting that these children can benefit from cognitive challenges regardless of the specific weight of external variables (Lidz, 1987). The moderate effect size observed suggests that the improvement in IQ is not merely attributable to measurement variability but reflects meaningful developmental change. Importantly, these findings support the idea that cognitive trajectories in autism are dynamic rather than fixed during early childhood, reinforcing the relevance of approaches that assess learning potential rather than static performance alone (Feuerstein et al., 1986; Haywood & Lidz, 2007).

However, the developmental pattern across specific cognitive processes was heterogeneous. While classification ability and perspective taking improved significantly over the two-year period, other processes such as auditory memory, visual memory, and sequential pattern completion remained relatively stable. Such variability highlights the multidimensional nature of cognitive development in ASD (American Psychiatric Association, 2014) and suggests that different cognitive domains may follow distinct developmental trajectories.

The improvement observed in classification ability may reflect the progressive development of conceptual organization and abstraction processes. Classification tasks require children to identify shared attributes and organize stimuli according to rules, processes that are closely related to fluid reasoning and early academic learning (Zacharov et al., 2021). Previous research has shown that individuals with ASD are capable of category learning, although the processes involved may differ from those observed in typically developing individuals (Mercado et al., 2020; Soulières et al., 2011; Wimmer et al., 2024). The present findings suggest that, within a structured learning environment, these conceptual abilities can show measurable developmental gains through mediated support during the preschool years (Gómez-Pérez et al., 2020).

Similarly, the increase observed in perspective-taking ability indicates progress in sociocognitive processes. Although social cognition is frequently described as a domain of difficulty in autism, growing evidence suggests that early social cognitive abilities can improve when children are exposed to enriched interactional contexts and guided learning experiences (Ni et al., 2021; Wood et al., 2019). In the present study, the perspective-taking task required children to coordinate verbal instructions with another person’s actions, a process that involves communicative monitoring, representational flexibility, and awareness of another individual’s informational needs. The developmental gains observed in this ability may therefore reflect the gradual integration of cognitive and communicative processes during early schooling.

The regression analyses provided further insight into the mechanisms underlying later cognitive outcomes. Visual memory emerged as the strongest predictor of intellectual functioning at age six, showing a higher beta coefficient than other variables and confirming its primary role in the model’s effect size. This finding is consistent with numerous studies indicating that visuospatial processing often represents a relative strength in individuals with ASD (Carmo et al., 2017; Stevenson et al., 2021). Visual memory supports the encoding and manipulation of visual information and may facilitate learning in contexts where information is structured spatially or presented through visual patterns (Forsberg et al., 2022; Soulières et al., 2011). Within educational settings, such strengths may enable children to rely on visual cues and spatial organization to scaffold learning (Tzuriel & Caspi, 2017).

This emphasizes the centrality of visuospatial processing as a key predictor in the cognitive profile of children with ASD. These non-verbal strengths appear to be more representative of their early learning potential than verbal or auditory measures, providing a more precise understanding of their developmental trajectory.

Sequential pattern completion also significantly predicted later cognitive ability. Pattern detection and rule extraction are closely associated with inductive reasoning and fluid intelligence (Zippert et al., 2021). Some theoretical frameworks propose that individuals with ASD demonstrate enhanced sensitivity to regularities and structured patterns in the environment (Morsanyi et al., 2019). This tendency may facilitate analytical reasoning in tasks involving sequences, rules, or structured visual information (Green et al., 2014). The present results are consistent with this perspective and suggest that pattern-based reasoning may play an important role in the cognitive learning pathways of children with ASD who display strong sensory engagement with visual and movement-based stimuli.

In addition to visuospatial abilities, classification ability remained a significant predictor of later intellectual functioning. This result underscores the importance of conceptual organization processes in early cognitive development. The ability to group stimuli based on shared attributes reflects emerging abstract thinking and the formation of conceptual categories, which are foundational for later academic skills (Soulières et al., 2011) such as mathematics and scientific reasoning.

Interestingly, perspective-taking ability also contributed significantly to the prediction of cognitive outcomes. This finding suggests that sociocognitive processes may play a broader role in intellectual development than traditionally assumed in autism research. Perspective taking may support learning by facilitating communication, collaborative problem-solving, and the interpretation of instructional cues. In classroom contexts, the ability to infer the intentions and informational needs of others may enhance engagement with guided learning activities (Tzuriel & Caspi, 2017), thereby indirectly contributing to cognitive growth.

In contrast, auditory memory and verbal planning did not significantly predict intellectual functioning in the final regression model. This pattern is consistent with previous literature suggesting that language-mediated working memory and verbally mediated executive processes may represent relative areas of difficulty in young children with ASD (Meir & Novogrodsky, 2020; Poulsen et al., 2024).

Auditory information must be processed sequentially and transiently, which may place greater demands on attentional control and linguistic processing (Gonçalves & Monteiro, 2023). Consequently, children may rely more heavily on visual strategies when solving cognitive tasks (Desaunay et al., 2020; Soulières et al., 2011).

Taken together, the results support the value of dynamic assessment approaches for understanding learning processes in autism (Musci & Brenlla, 2017; Tzuriel & Caspi, 2017). Unlike traditional static measures of intelligence, dynamic assessment evaluates the learner’s responsiveness to mediated support and the capacity for cognitive change (Daneshfar & Moharami, 2018; Kee, 2016). In the present study, the EHPAP tasks captured modifiable cognitive processes that were predictive of later intellectual functioning, demonstrating the potential of such approaches to identify early learning pathways (Luković et al., 2022; Valencia-Naranjo & Robles-Bello, 2017).

From an applied perspective, the findings provide preliminary insights that may help inform early intervention and educational practice. These exploratory results suggest that interventions that incorporate visual supports, pattern-based learning activities, and structured categorization tasks could potentially align with the cognitive strengths observed in many children with ASD (Camino-Alarcón et al., 2024). In addition, supporting early sociocognitive skills such as perspective taking may enhance not only social functioning but also broader learning outcomes by improving engagement in collaborative and guided learning contexts (Kee, 2016). Crucially, these predictors remained significant even after accounting for baseline IQ, confirming their unique contribution to the developmental trajectory. While the variance explained (*R*^2^ = 0.51), it should be interpreted with caution, as the small sample size may increase the risk of multicollinearity and limit the stability of the predictive coefficients; the theoretical grounding of the model and the consistent effect sizes support the relevance of these findings.

Furthermore, the study highlights the potential relevance of sensory processing profiles for understanding cognitive development in autism. Children in the present sample displayed a seeker–sensitive sensory pattern characterized by active sensory exploration combined with relatively preserved self-regulation (Dunn, 2016). The consistency of sensory scores across the sample was verified, showing minimal dispersion and supporting the use of a homogeneous profile for this cohort. While the direct predictive link between sensory profiles and cognitive outcomes was not statistically modeled, the alignment between these variables offers a plausible theoretical framework for future research. These findings indicate a significant association between sensory patterns and cognitive abilities rather than a causal relationship, suggesting that sensory engagement and visuospatial reasoning may develop in tandem during early childhood. Furthermore, in the absence of a control group, these associations should be interpreted as a specific characterization of this cohort rather than a unique feature of the ASD cognitive profile. While sensory processing was not formally included as a predictor in the regression models, its qualitative alignment with the identified cognitive strengths provides a contextual baseline for interpreting the developmental outcomes of this sample. Such a profile may encourage engagement with visually structured and movement-based experiences, which in turn may support the development of visuospatial reasoning and pattern detection skills (Fernández-Pires et al., 2020; Bazelmans et al., 2021). Future research should continue to examine how sensory processing characteristics interact with cognitive development to shape individualized learning trajectories.

Overall, the findings suggest that early cognitive development in autism is shaped by a complex interplay between sensory experiences, domain-specific cognitive processes, and opportunities for mediated learning (Vygotsky, 1978). Integrating these dimensions may provide a more comprehensive framework for understanding developmental variability within the autism spectrum.

### Limitations

Several limitations should be considered when interpreting the findings of this study:

First, the relatively small sample size (N = 44) limits statistical power and restricts the generalizability of the results. This small N may also increase the risk of model overfitting in the regression analyses; therefore, the reported multiple correlation (*R*^2^ = 0.51) should be interpreted with caution, as regression coefficients in small samples can be susceptible to fluctuations. Future research with larger, more diverse cohorts is necessary to stabilize these estimates and confirm the predictive power of the identified variables. Furthermore, a key limitation of this study is the absence of a control group (either receiving an alternative intervention or no intervention). Consequently, the observed cognitive and developmental gains cannot be unequivocally isolated from the broader effects of the ECI program or general maturation processes. Future research should implement controlled designs to address this gap.

Second, the sample consisted exclusively of boys with ASD. This is a significant limitation, as the female phenotype in autism often involves different sensory reactivity, including greater internal sensitivity that may not be behaviorally visible, and “camouflaging” strategies that could influence cognitive learning pathways differently (Hull et al., 2017; Lai et al., 2015; Rynkiewicz et al., 2016).

Third, the study focused on children with a sensory-seeking profile. While this increased sample homogeneity, it prevents comparisons with other profiles (e.g., sensory avoiders). Therefore, the results should be viewed as a characterization of this specific subgroup rather than a universal or causal relationship between sensory processing and intelligence.

Fourth, the participants were recruited from a single Early Childhood Intervention (ECI) center. This may limit the external validity of the findings and means that the potential influence of the specific intervention received was not independently controlled. Consequently, observed cognitive gains may reflect a combination of the cognitive predictors identified and the effects of concurrent therapeutic support, as well as the shared educational context.

Finally, although the longitudinal design allowed observation of change, only two assessment points were included. Designs with multiple assessment timepoints would better capture complex developmental trajectories and the influence of early intervention.

## 5. Conclusions

The present study provides preliminary evidence of a preferential developmental pathway for children with ASD who exhibit high sensory engagement (seeker profile). The findings suggest that, for this subgroup, visuospatial cognitive processes—particularly visual memory and pattern-based reasoning—constitute a relevant cognitive pathway associated with intellectual development. In this context, active engagement with visual and movement stimuli may function as a ‘scaffold’ for acquiring more complex reasoning skills, rather than representing a universal centrality. This underscores the importance of personalized educational strategies that capitalize on these specific sensory strengths. Consequently, learning potential and cognitive functioning show measurable developmental changes during the preschool years in children with autism spectrum disorder who present a seeker–sensitive sensory processing profile.

The findings suggest that visuospatial cognitive processes, particularly visual memory and pattern-based reasoning, suggest a preferential developmental pathway for children with ASD and high sensory engagement, where visuospatial processing and pattern detection act as a ‘scaffold’ for acquiring more complex reasoning skills. These results align with previous research indicating that visual processing may represent a relative strength in many children with autism.

Importantly, the use of dynamic assessment allowed identification of modifiable cognitive processes that may support learning and developmental progress. From an applied perspective, early educational interventions that capitalize on visuospatial strengths and structured pattern-based learning strategies may be particularly beneficial for this population.

Overall, integrating sensory processing profiles with dynamic cognitive assessment may represent a promising approach for identifying early learning pathways and designing more individualized intervention strategies for children with autism spectrum disorder.

## Figures and Tables

**Table 1:** Average scores by sensory quadrant presence (raw scores).

Domain	Section/Quadrant	Percentile	Interpretation
Sensory Quadrants	Low Registration	35	Perceives most stimuli, with a slight tendency to become distracted if they are not very pronounced.
	Sensory Seeking	65	High exploratory sensory engagement.
	Sensory Sensitivity	45	Within typical range.
	Sensory Avoiding	40	Within typical range. May move away from loud noises or group activities, but without significant interference.
Sensory Sections	Auditory	40	He is sometimes bothered by unexpected noises, but he tolerates the school environment well.
	Visual	65	High visual engagement.
	Tactile	45	Slightly sensitive to certain textures or physical contact, but functional.
	Movement (Proprioceptive/Vestibular)	62	Moderate proprioceptive seeking.
	Oral	50	Normal; no significant atypical oral behaviors.
Behavioral Sections	Social behavior	55	Appropriate interaction; may be somewhat rigid when taking turns or discussing topics.
	Attention	50	Good attention span if the topic interests them; slightly distracted in noisy environments.
	Autonomy/Self-control	57	Regulates their behavior well; occasional difficulties with unexpected changes.
	Emotional response	55	Handles emotions appropriately, although needs support when faced with frustration.

**Table 2:** Descriptive statistics and between-group comparison in study participants (n = 44) according to age.

	4 Years	6 Years	Between Group	
	M (SD)	M (SD)	t	Cohen’s d
IQ (General cognitive ability)	100.2 (15)	106.7 (20.6)	2.03 *	0.53
EHPAP				
Classification	51.2 (19.2)	57.3 (22.6)	3.13 *	0.49
Auditory Memory	48.9 (22.6)	51.3 (24.8)	0.28 ^ns^	0.26
Visual Memory	74 (11.5)	75.2 (18.1)	0.37 ^ns^	0.08
Sequential Pattern Completion	90.1 (16.6)	92.3 (11.2)	0.73 ^ns^	0.16
Verbal Planning	49.18 (20.2)	52.7 (26.3)	0.70 ^ns^	0.15
Perspective Taking	52.7 (26.4)	57.9 (22.4)	1.90 *	0.39

t = t students; d = Cohen’s d effect size; * *p* < 0.05; ^ns^ = not significant. Note: EHPAP scores are reported as raw scores, while IQ scores are standardized.

**Table 3:** Values of the regression equation for the VIs for child with ASD (6 years).

Predictor	B	SE B	β	t	*p*	Tolerance	VIF
Step 1							
Classification	0.21	0.09	0.31	2.29	.027		
Auditory Memory	0.10	0.08	0.17	1.21	.233		
Model statistics							
*R*^2^ = 0.18	F(2,41) = 4.47	*p* = .017					
Step 2							
Classification	0.18	0.08	0.26	2.11	.041		
Auditory Memory	0.07	0.07	0.11	0.98	.332		
Visual Memory	0.35	0.11	0.42	3.18	.003		
Sequential Pattern Completion	0.18	0.07	0.29	2.45	.019		
Model statistics							
*R*^2^ = 0.42	Δ*R*^2^ = 0.24	F(4,39) = 7.03	*p* < .001				
Step 3							
Classification	0.16	0.07	0.23	2.02	.049	0.62	1.61
Auditory Memory	0.06	0.07	0.09	0.82	.417	0.68	1.47
Visual Memory	0.33	0.10	0.40	3.11	.003	0.59	1.70
Sequential Pattern Completion	0.17	0.07	0.27	2.36	.024	0.64	1.55
Perspective Taking	0.14	0.06	0.26	2.12	.040	0.71	1.40
Verbal Planning	0.09	0.08	0.15	1.08	.286	0.66	1.52
Final model							
*R*^2^ = 0.51	Adjusted R^2^ = 0.44	F(6,37) = 6.42	*p* < 0.001				

## Data Availability

The data presented in this study are available on request from the corresponding author. The data are not publicly available due to ethical reasons for confidentiality. The data are transcripts of interviews.

## Abbreviations

The following abbreviations are used in this manuscript:

ASD	Autism spectrum disorder
ZPD	Zone of Proximal Development
MLE	Mediated Learning Experience
EHPAP	Escala de Habilidades y Potencial de Aprendizaje en Preescolares
